# Corrigendum: Paenibacillus melissococcoides sp. nov., isolated from a honey bee colony affected by European foulbrood disease

**DOI:** 10.1099/ijsem.0.006408

**Published:** 2024-06-07

**Authors:** Florine Ory, Vincent Dietemann, Anne Guisolan, Ueli von Ah, Charlotte Fleuti, Emilie Michellod, Olivier Schumpp, Simone Oberhaensli, Jean-Daniel Charrière, Benjamin Dainat

**Affiliations:** 1 Swiss Bee Research Centre, Agroscope, Bern, Switzerland; 2 Department Ecology and Evolution, University of Lausanne, Lausanne, Switzerland; 3 Biotechnology Research Group, Agroscope, Bern, Switzerland; 4 Methods Development and Analytics, Agroscope, Bern, Switzerland; 5 Virology, Bacteriology and Phytoplasmology, Agroscope, Nyon, Switzerland; 6 Interfaculty Bioinformatics Unit and SIB Swiss Institute of Bioinformatics, University of Bern, Bern, Switzerland

In the published version of this article, the author list, affiliations and author contributions were incorrectly listed as:

**Author list**

Florine Ory^1^, Vincent Dietemann^1,2^, Anne Guisolan^3^, Ueli von Ah^3^, Charlotte Fleuti^4^, Simone Oberhaensli^5^, Jean- Daniel Charrière^1^ and Benjamin Dainat^1^

^1^Swiss Bee Research Centre, Agroscope, Bern, Switzerland; ^2^Department Ecology and Evolution, University of Lausanne, Lausanne, Switzerland; ^3^Biotechnology Research Group, Agroscope, Bern, Switzerland; ^4^Methods Development and Analytics, Agroscope, Bern, Switzerland; ^5^Interfaculty Bioinformatics Unit and SIB Swiss Institute of Bioinformatics, University of Bern, Bern, Switzerland.

**Author Contribution**

F.O.: conceptualization, investigation, formal analysis, validation, visualization, writing – original. V.D.: conceptualization, supervision, project administration, validation, visualization, writing – review and editing. A.G: investigation, formal analysis. U.v.A.: investigation, formal analysis. C.F: investigation, formal analysis, writing – review and editing S.O: investigation, formal analysis, writing – review and editing. J.D.C.: project administration, validation, writing – review and editing. B.D.: conceptualization, investigation, supervision, project administration, validation, visualization, writing – review and editing.

The author list, affiliations and author contribution should have been listed as:

**Author List**

Florine Ory^1^, Vincent Dietemann^1,2^, Anne Guisolan^3^, Ueli von Ah^3^, Charlotte Fleuti^4^, Emilie Michellod^5^, Olivier Schumpp^5^, Simone Oberhaensli^6^, Jean-Daniel Charrière^1^ and Benjamin Dainat^1^.

^1^Swiss Bee Research Centre, Agroscope, Bern, Switzerland; ^2^Department Ecology and Evolution, University of Lausanne, Lausanne, Switzerland; ^3^Biotechnology Research Group, Agroscope, Bern, Switerland; ^4^Methods Development and Analytics, Agroscope, Bern, Switzerland; ^5^Virology, Bacteriology and Phytoplasmology, Agroscope, Nyon, Switzerland; ^6^Interfaculty Bioinformatics Unit and SIB Swiss Institute of Bioinformatics, University of Bern, Bern, Switzerland.

**Author Contribution**

F.O.: conceptualization, investigation, formal analysis, validation, visualization, writing – original. V.D.: conceptualization, supervision, project administration, validation, visualization, writing – review and editing. A.G: investigation, formal analysis. U.v.A.: investigation, formal analysis. C.F.: investigation, formal analysis, writing – review and editing. E.M.: investigation. O.S.: methodology, formal analysis. S.O.: investigation, formal analysis, writing – review and editing. J.D.C.: project administration, validation, writing – review and editing. B.D.: conceptualization, investigation, supervision, project administration, validation, visualization, writing – review and editing.

The authors apologise for any inconvenience caused.

